# Complete genome sequence of *Bradyrhizobium lupini* strain BA2, isolated from root nodules of *Retama dasycarpa*

**DOI:** 10.1128/mra.00834-25

**Published:** 2025-10-10

**Authors:** Mouad Lamrabet, Zohra Chaddad, Soufiane Alami, Mustapha Missbah El Idrissi

**Affiliations:** 1 Laboratory of Microbiology and Molecular Biology, Faculty of Sciences, Mohammed V University in Rabat 107736, Rabat, Morocco; University of Maryland School of Medicine, Baltimore, Maryland, USA

**Keywords:** *Bradyrhizobium lupini*, *Retama dasycarpa*, root nodules

## Abstract

In this study, we report the complete genome of *Bradyrhizobium lupini* strain BA2, isolated from *Retama dasycarpa* root nodules. As the only high-quality complete genome of *B. lupini* in public databases, this assembly serves as the species reference.

## ANNOUNCEMENT

*Bradyrhizobium* spp. are Gram-negative bacteria that fix atmospheric nitrogen in symbiosis with leguminous plants (1). *Bradyrhizobium lupini* strain BA2 was isolated from nodules of *Retama dasycarpa* growing in the Ait Benammar region of the High Atlas Mountains, Morocco (31°38′96.16″N, 7°40′86.57″W) (2). Nodules were surface-sterilized with HgCl_2_ and ethanol, crushed, and streaked onto yeast extract-mannitol agar (YEM). Plates were incubated at 28°C for 10 days, and pure cultures were obtained through repeated subculturing.

The strain BA2 appears as an efficient nitrogen-fixing bacteria that promote plant growth-promoting (PGP) activities and exhibits high tolerance to water stress (2, 3). It was previously identified as *B. lupini* USDA 3051^T^ based on multi-locus sequence analysis (2). In this work, we present the complete genome sequence of *B. lupini* BA2, which will provide valuable insights into the genomic features involved in the symbiosis, stress tolerance, and plant growth-promoting traits.

Genomic DNA (gDNA) was extracted from a 5-day bacterial culture incubated in TY liquid medium at 28°C (4), using the PureLink Genomic DNA miniKit (Invitrogen by Life Technologies). The library was prepared using the Rapid Barcoding Sequencing Kit (SQK-RBK004) and sequenced on the GridION x5 device, using FLO-MIN106 flowcell (Oxford Nanopore Technologies, Oxford, UK). Base calling was performed using MinKNOW software (v. 24.02.16) with high-accuracy base calling mode in Dorado software (version 7.3.11). The quality of the generated reads was examined using LongQC software (v. 1.2.1) (5), and the low-quality reads and adapter were trimmed and filtered using Porechop (v. 0.2.4) (6), and nanofilt (v. 2.8.0) (7), respectively. *De novo* assembly was performed using Flye (v. 2.9.2) (8), which assessed circularity by detecting overlapping ends in the assembly graph. Circular sequences were rotated with Circlator v1.5.5 (9), which placed the replication initiator gene (*dnaA*) at nucleotide position 1 on the chromosome and oriented the plasmid at its respective replication-associated genes. Then, the genome sequences were sequentially corrected using racon (v. 1.5.0) (10) and polished by Medaka (v. 1.11.3) (11) to generate consensus sequences. The completeness of the assemblies was assessed using BUSCO (v. 5.7.1) (12) and checked using Quast (v. 5.2.0) (13). The assembled genome was annotated via NCBI Prokaryotic Genome Annotation Pipeline (PGAP) (14), and the average nucleotide identity (ANI) values were calculated using Pyani software v. 0.2.12 (ANIb method) (15) against closely related type and representative genomes obtained from NCBI RefSeq. Digital DNA-DNA hybridization (dDDH) was computed with GGDC 3.0 (16), using formula 2. Default parameters were used for all software.

**Fig 1:**
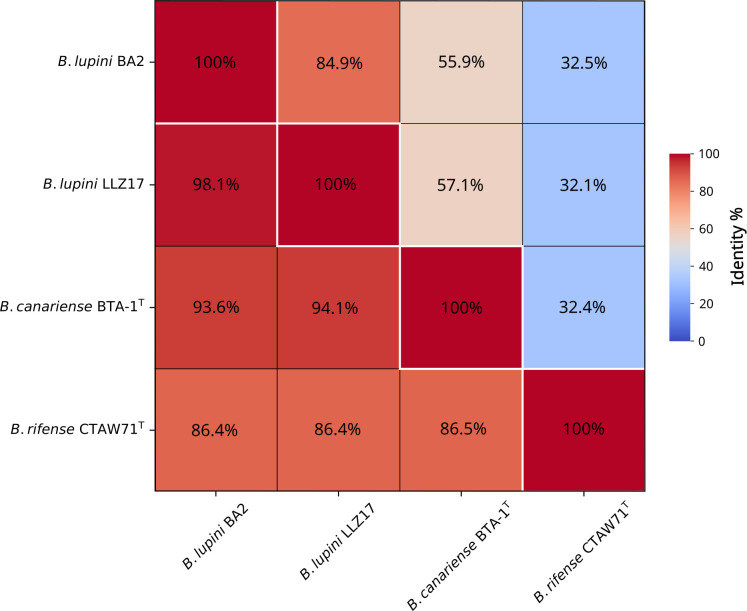
Comparative genomic analysis of *Bradyrhizobium lupini* BA2 and closely related type strains. The heatmap displays pairwise average nucleotide identity (ANI) percentages (lower triangle) and digital DNA-DNA hybridization (dDDH) values (upper triangle).

The complete genome assembly comprises two circular contigs: a chromosome (7,971,975 bp; 63% GC) and a plasmid (495,480 bp; 61.5% GC), with a sequencing coverage of 64×. Annotation identified 8,100 genes, including 7,532 protein-coding sequences (CDSs) and 55 RNA genes (Table 1). Phylogenomic analysis showed 98.4% ANI and 84.9% dDDH with the closest relative, *B. lupini* LLZ14 (GCA_040939785.1), exceeding species thresholds (95%–96% ANI, 70% dDDH) (17) (Fig. 1). These results confirm the classification of strain BA2 as *B. lupini* and establish it as a high-quality genomic reference for future studies on this species.

**TABLE 1:** Genome features of *Bradyrhizobium lupini* strain BA2

Parameter	Value for *Bradyrhizobium lupini* BA2
No. of raw reads	18,904
No. of cleaned reads	17,810
Mean read length (bp)	10,330
Read N50 (bp)	18,805
No. of contigs	2
Genome length (bp)	8,467,455
Coverage (×)	64
Chromosome length (bp)	7,971,975
Plasmid length (bp)	495,480
GC content (%)	63.08
No. of CDSs	7,532
No. of rRNAs	3
No. of tRNAs	48
No. of ncRNAs	4
BioSample ID	SAMN43942329
BioProject ID	PRJNA1165669
NCBI RefSeq assembly	GCF_042691665.1

## Data Availability

The complete genome sequence of *Bradyrhizobium lupini* strain BA2 is available in the NCBI GenBank database under the accession number GCA_042691665.1 with the BioProject accession number PRJNA1165669 and BioSample accession number SAMN43942329. The raw sequencing reads are available in the Sequence Read Archive (SRA) under the accession number SRR34821080.

## References

[1] Ormeño-Orrillo E, Martínez-Romero E. 2019. A genomotaxonomy view of the Bradyrhizobium genus. Front Microbiol 10:1334. doi:10.3389/fmicb.2019.01334 31263459 PMC6585233

[2] Lamrabet M, Chaddad Z, Bouhnik O, Alami S, Kaddouri K, Bennis M, Lamin H, Mnasri B, Bourgerie S, Morabito D, Abdelmoumen H, Bedmar EJ, Missbah El Idrissi M. 2023. Different species of Bradyrhizobium from symbiovars genistearum and retamae nodulate the endemic Retama dasycarpa in the High Atlas Mountains. FEMS Microbiol Ecol 99:fiad038. doi:10.1093/femsec/fiad038 37019822

[3] Lamrabet M, Chaddad Z, Bouhnik O, Kaddouri K, Alami S, Bennis M, Mnasri B, Abdelmoumen H, El Idrissi MM. 2024. Effect of native plant growth promoting osmotolerant Bradyrhizobium strains on drought stress tolerance in Retama dasycarpa. Agric, Ecosyst Environ, Appl Soil Ecol 203:105662. doi:10.1016/j.apsoil.2024.105662

[4] Beringer JE. 1974. R factor transfer in Rhizobium leguminosarum. Microbiology (Reading, Engl) 84:188–198. doi:10.1099/00221287-84-1-188 4612098

[5] Fukasawa Y, Ermini L, Wang H, Carty K, Cheung MS. 2020. LongQC: a quality control tool for third generation sequencing long read data. G3 Bethesda Md 10:1193–1196. doi:10.1534/g3.119.400864 32041730 PMC7144081

[6] Wick RR, Judd LM, Gorrie CL, Holt KE. 2017. Completing bacterial genome assemblies with multiplex MinION sequencing. Microb Genom 3:e000132. doi:10.1099/mgen.0.000132 29177090 PMC5695209

[7] De Coster W, D’Hert S, Schultz DT, Cruts M, Van Broeckhoven C. 2018. NanoPack: visualizing and processing long-read sequencing data. Bioinformatics 34:2666–2669. doi:10.1093/bioinformatics/bty149 29547981 PMC6061794

[8] Kolmogorov M, Yuan J, Lin Y, Pevzner PA. 2019. Assembly of long, error-prone reads using repeat graphs. Nat Biotechnol 37:540–546. doi:10.1038/s41587-019-0072-8 30936562

[9] Hunt M, Silva ND, Otto TD, Parkhill J, Keane JA, Harris SR. 2015. Circlator: automated circularization of genome assemblies using long sequencing reads. Genome Biol 16:294. doi:10.1186/s13059-015-0849-0 26714481 PMC4699355

[10] Vaser R, Sović I, Nagarajan N, Šikić M. 2017. Fast and accurate de novo genome assembly from long uncorrected reads. Genome Res 27:737–746. doi:10.1101/gr.214270.116 28100585 PMC5411768

[11] Sahlin K, Lim MCW, Prost S. 2021. NGSpeciesID: DNA barcode and amplicon consensus generation from long-read sequencing data. Ecol Evol 11:1392–1398. doi:10.1002/ece3.7146 33598139 PMC7863402

[12] Manni M, Berkeley MR, Seppey M, Zdobnov EM. 2021. BUSCO: assessing genomic data quality and beyond. Curr Protoc 1:e323. doi:10.1002/cpz1.323 34936221

[13] Mikheenko A, Prjibelski A, Saveliev V, Antipov D, Gurevich A. 2018. Versatile genome assembly evaluation with QUAST-LG. Bioinformatics 34:i142–i150. doi:10.1093/bioinformatics/bty266 29949969 PMC6022658

[14] Tatusova T, DiCuccio M, Badretdin A, Chetvernin V, Nawrocki EP, Zaslavsky L, Lomsadze A, Pruitt KD, Borodovsky M, Ostell J. 2016. NCBI prokaryotic genome annotation pipeline. Nucleic Acids Res 44:6614–6624. doi:10.1093/nar/gkw569 27342282 PMC5001611

[15] Pritchard L, Glover RH, Humphris S, Elphinstone JG, Toth IK. 2016. Genomics and taxonomy in diagnostics for food security: soft-rotting enterobacterial plant pathogens. Anal Methods 8:12–24. doi:10.1039/C5AY02550H

[16] Meier-Kolthoff JP, Carbasse JS, Peinado-Olarte RL, Göker M. 2022. TYGS and LPSN: a database tandem for fast and reliable genome-based classification and nomenclature of prokaryotes. Nucleic Acids Res 50:D801–D807. doi:10.1093/nar/gkab902 34634793 PMC8728197

[17] Chaddad Z, Bouhnik O, Lamrabet M, Alami S, Missbah El Idrissi M. 2025. Complete genome sequence of Bradyrhizobium lupini LLZ14, a nitrogen-fixing and plant growth-promoting bacterium. Microbiol Resour Announc 14:e0093524. doi:10.1128/mra.00935-24 39878511 PMC11895481

